# Physiological Sympathetic Activation Reduces Systemic Inflammation: Role of Baroreflex and Chemoreflex

**DOI:** 10.3389/fimmu.2021.637845

**Published:** 2021-04-28

**Authors:** Fernanda Brognara, Jaci Airton Castania, Alexandre Kanashiro, Daniel Penteado Martins Dias, Helio Cesar Salgado

**Affiliations:** ^1^ Department of Physiology, Ribeirão Preto Medical School, University of São Paulo, Ribeirão Preto, Brazil; ^2^ Department of Neuroscience and Behavior, Ribeirão Preto Medical School, University of São Paulo, Ribeirão Preto, Brazil; ^3^ Barão de Mauá University Center, Ribeirão Preto, Brazil

**Keywords:** baroreceptors, bilateral carotid occlusion, chemoreceptors, inflammation, neuroimmune interactions, sympathetic activation

## Abstract

Baroreflex and chemoreflex act through the autonomic nervous system, which is involved with the neural regulation of inflammation. The present study reports the effects of reflex physiological sympathetic activation in endotoxemic rats using bilateral carotid occlusion (BCO), a physiological approach involving the baroreflex and chemoreflex mechanisms and the influence of the baroreceptors and peripheral chemoreceptors in the cardiovascular and systemic inflammatory responses. After lipopolysaccharide (LPS) administration, the arterial pressure was recorded during 360 min in unanesthetized rats, and serial blood samples were collected to analyze the plasma cytokine levels. BCO elicited the reflex activation of the sympathetic nervous system, providing the following outcomes: (I) increased the power of the low-frequency band in the spectrum of the systolic arterial pressure during the BCO period; (II) reduced the levels of pro-inflammatory cytokines in plasma, including the tumor necrosis factor (TNF) and the interleukin (IL)-1*β*; (III) increased the plasma levels of anti-inflammatory cytokine IL-10, 90 min after LPS administration. Moreover, selective baroreceptor or chemoreceptor denervation deactivated mechanosensitive and chemical sensors, respectively, and decreased the release of the LPS-induced cytokine but did not alter the BCO modulatory effects. These results show, for the first time, that physiological reflex activation of the sympathetic circuit decreases the inflammatory response in endotoxemic rats and suggest a novel function for the baroreceptors as immunosensors during the systemic inflammation.

## Introduction

The interaction between the central nervous system and the immune system has been studied since the 19th century (1). Of note, this interaction plays a fundamental role in the regulation of inflammation *via* activation of neuroendocrine circuits including the hypothalamic–pituitary–adrenal axis (2, 3), the “cholinergic anti-inflammatory pathway” (4, 5), and the “splanchnic anti-inflammatory pathway” (6). The autonomic nervous system has been the focus of studies involving control of the immune system (6–12). These neuroimmune circuits have been described in endotoxemia, the most known model of inflammation. Lipopolysaccharide (LPS), also known as endotoxin, is a molecule present in the cell wall of Gram-negative bacteria and, when administrated, can stimulate innate immunity *via* activation of Toll-like receptor 4 (TLR-4) expressed on the surface of macrophages, for example, inducing the release of pro-inflammatory cytokines (13–15). Besides, in rodents, the outer membrane LPS of Gram-negative bacteria can also activate the noncanonical inflammasome pathway and stimulate the release of IL-1β *via* caspase-11 (16, 17). Briefly, intracellular LPS is recognized by the CARD domain of caspase-11, which cleaves the pore-forming protein gasdermin D (17–19). Through a K^+^ efflux-dependent process, the N-terminal domain of gasdermin D activates NLRP3 inflammasome, which triggers the release of IL-1β by active caspase-1, increasing the inflammation (18).

The body has physiological reflex mechanisms to activate or deactivate both the sympathetic and/or the parasympathetic branches. Among these mechanisms are: (I) the baroreflex, which provides the moment-to-moment control of arterial pressure mediated primarily by the arterial baroreceptors, which, when stimulated, activate the parasympathetic branch and inhibit the sympathetic branch; and (II) the chemoreflex, which maintains the cardiorespiratory homeostasis in response to changes in blood gas concentrations (such as oxygen or carbon dioxide) due to the presence of the peripheral chemoreceptors, which, when stimulated, activate both branches of the autonomic nervous system, *i.e.*, the sympathetic and the parasympathetic. Thus, physiological methods able to activate or deactivate the autonomic nervous system reflexively could be useful to mimic the authentic role of this system during inflammation. However, to the best of our knowledge, no previous study has used a methodological approach that activates the autonomic nervous system reflexively, during a reliable physiological context, so as to investigate the importance of the nervous system during an immune challenge.

The bilateral carotid occlusion (BCO) is one of the techniques used to elicit global reflex activation of the sympathetic nervous system in both unanesthetized (20–23) and anesthetized animals (24, 25). Moreover, when the common carotids arteries are occluded temporarily, there is a significant reduction in the arterial pressure and blood flow inside the carotid sinus region, culminating with the deactivation of the carotid baroreceptors. This inactivation of the carotid baroreceptors induces an increase in sympathetic activity to the blood vessels, increasing the global peripheral resistance, combined with a concomitant reduction in cardiac parasympathetic activity, determining the increase in arterial pressure (26, 27). In addition, there is the activation of the carotid chemoreceptors, due to the hypoxia caused by the reduction of blood flow into the carotid sinus, contributing to the increase in sympathetic activity (26, 27).

Because the BCO is a methodological approach that promotes reflex sympathetic activation, it could be used to study the influence of the autonomic nervous system in the control of systemic inflammation. Furthermore, taking into account that the baroreceptors and the chemoreceptors are involved in the pressor response to BCO, it is also necessary to examine the individual participation of these receptors during an inflammatory process. Thus, the present study aimed to investigate in unanesthetized LPS-induced endotoxemic rats: (I) the effect of BCO-induced sympathetic reflex activation; (II) the importance of both the baroreflex and the chemoreflex in this procedure; and (III) the influence of the baroreceptors and the peripheral chemoreceptors, through their specific surgical denervation, in the systemic inflammatory response.

## Methods

### Experimental Animals and Groups

Male Wistar-Hannover rats (250–320 g) obtained from the Main Animal Facility of the University of São Paulo (Campus of Ribeirão Preto; Ribeirão Preto, SP, Brazil) were used. The animals were maintained in individual cages under controlled temperature (22°C), and a constant 12-hour light–dark cycle, with free access to water and food. All procedures were reviewed and approved by the Committee of Ethics in Animal Research of the Ribeirão Preto Medical School - University of São Paulo (Protocol # 194/2016). The rats were divided into seven experimental groups:

- **Saline (n = 9):** fictitious surgery and saline administration.
- **LPS (n = 8):** bilateral implantation of the pneumatic cuffs, without BCO, with the administration of LPS.
- **BCO + LPS (n = 8):** bilateral implantation of the pneumatic cuffs and, BCO during 20 s, with the administration of LPS.
- **BARO-X + LPS (n = 7):** selective denervation of the aortic and carotid baroreceptors, bilateral implantation of the pneumatic cuffs, without BCO, with the administration of LPS.
- **BARO-X + BCO + LPS (n = 9):** selective denervation of the aortic and carotid baroreceptors, bilateral implantation of the pneumatic cuffs and BCO during 20 s, with the administration of LPS.
- **CHEMO-X + LPS (n = 7):** selective denervation of the carotid chemoreceptors, bilateral implantation of the pneumatic cuffs, without BCO, with the administration of LPS.
- **CHEMO-X + BCO + LPS (n = 9):** selective denervation of the carotid chemoreceptors, bilateral implantation of the pneumatic cuffs and BCO during 20 s, with the administration of LPS.

### Surgical Procedures

The animals were anesthetized with a mixture of Ketamine and Xylazine (50 mg/kg and 10 mg/kg, i.p.) and then subjected to the surgical procedure for cannulation of the left femoral artery and vein for arterial pressure recording and LPS (or saline) administration, respectively. Briefly, polyethylene tubes (PE-50 soldered to PE-10 polyethylene tube; Intramedic, Clay Adams, Parsippany, NJ, USA) were implanted into the femoral artery and vein and pulled up through a subcutaneous track to the rat’s neck and exteriorized in the nape. The catheter inserted into the femoral artery was filled with 100 IU/ml heparin in saline. In the same surgery, except for the saline group, all other animals had pneumatic cuffs implanted, bilaterally, around the common carotid arteries. For this procedure, an anterior median cervicotomy was carried out, while the sternohyoid and sternocleidomastoid muscles were identified and retracted, exposing the common carotid artery and the carotid sinus. To implant the pneumatic cuffs, the common carotid arteries were carefully isolated, and the pneumatic cuffs fixed around them with cotton threads. The catheters for filling the balloons were exteriorized and fixed onto the back of the neck, as well as the vascular catheters. In the saline group, the animals were subjected to the same surgical procedures, but the pneumatic cuffs were not implanted around the common carotid arteries. The surgical incisions were properly sutured and, immediately after the surgery, an analgesic was administered (tramadol hydrochloride, 2 mg/kg, s.c.). Pneumatic cuffs (**Supplementary Figures 1A–C**) were made following the technique described by Maio et al. (28).

Concerning the groups IV and V, in the same surgery for the pneumatic cuff implantation, the rats were submitted to procedures for baroreceptor denervation. This approach was undertaken to prevent the attenuation of the sympathetic activity by the baroreceptors during the elevation of the arterial pressure resulting from the BCO, and also to eliminate a possible influence of the baroreflex in the anti-inflammatory response caused by sympathetic activation. For this purpose, the denervation of the aortic and carotid baroreceptors was performed bilaterally. The carotid baroreceptor denervation was performed according to the technique described by Castania et al. (29). Briefly, the common carotid region and the carotid bifurcation were exposed. Next to the glossopharyngeal nerve, two branches were identified, usually separated by a small artery. With the aid of a magnifying glass one of these branches, which carries the afferent fibers of the carotid baroreceptors, was carefully sectioned. The aortic baroreceptor denervation, on the other hand, was carried out following the technique described by Krieger (30), in which the superior laryngeal nerve and the superior cervical ganglion were isolated and sectioned. The cervical sympathetic trunk was also sectioned caudally to the superior cervical ganglion, which was dissected and removed. The procedure was performed on both sides. On the other hand, groups VI and VII were submitted to the denervation of the carotid chemoreceptors so as to study their influences in the inflammatory response during the BCO. The technique was also performed bilaterally, following the method described by Franchini and Krieger (31). For this procedure, the common carotid artery and its bifurcation were exposed. With the aid of a magnifying glass, the carotid body was identified, and the carotid body artery was carefully isolated and sectioned distally to the ligature.

### Arterial Pressure Recording

After recovering from the surgery, which took 24 h, the unanesthetized rats were connected to the arterial pressure recording system. Briefly, the arterial catheter was connected to a pressure transducer (MLT844; ADInstruments, Bella Vista, Australia), and the signal was amplified (ML224; ADInstruments, Bella Vista, Australia) and sampled at 2 kHz by an IBM/PC computer (Core 2 Duo, 2.2 GHz, 4 GB RAM) attached to an analog-to-digital interface (PowerLab, ADInstruments, Bella Vista, Australia). The experiment was conducted with the animals moving freely in their own cage, and silence was maintained to minimize environmental stress. Arterial pressure recordings were processed with computer software (LabChart 7.0, ADInstruments, Bella Vista, Australia) capable of detecting inflection points and generate mean arterial pressure, systolic arterial pressure, diastolic arterial pressure, and heart rate beat-by-beat time series. In the case of groups with BCO, the two catheters for filling the balloons were connected to a syringe with water through a personalized Y-shaped polyethylene tubing to perform the occlusion simultaneously on both sides.

Following the basal recording of pulsatile arterial pressure during 30 min, both common carotid arteries were occluded for 20 s, decreasing the blood flow above the carotid region (**Supplementary Figures 1D, E**). Next, the balloons were deflated to re-establish the blood flow. Immediately after the end of the occlusion, LPS [0.06 mg/kg (i.v.); *Escherichia coli*-0111: B4 purified by phenol extraction; Sigma-Aldrich, St. Louis, MO, USA] was administered (**Supplementary Figure 1F**). For the other groups not subjected to BCO, saline or LPS was administered immediately after the end of the basal recordings. Arterial pressure was recorded continuously during 360 min after the administration of LPS or saline, in all groups (**Supplementary Figure 1F**). During this period, serial blood samples (250 µl per sample) were taken at 90, 180, 270, and 360 min after the administration of LPS or saline, through the catheter placed into the left femoral artery (**Supplementary Figure 1F**). Blood samples were collected with heparin and kept on ice until centrifuged at 4°C for 15 min at 5,000 rpm. Subsequently, the plasma was collected and stored at −80°C until processing. In the groups with denervation (baroreceptor or chemoreceptor), at the end of the last blood collection, tests were performed with phenylephrine (2 µg in 0.1 ml, i.v.) and potassium cyanide (40 µg in 0.1 ml, i.v.) to confirm the correct denervation of each animal. Subjects that were not adequately denervated were not included in the study.

### Cardiocirculatory Variability Analysis

Beat-by-beat time series with systolic arterial pressure and cardiac interval values were extracted from periods of approximately 10 min for each moment (basal, 90, 180, 270, and 360 min after LPS or saline) from pulsatile arterial pressure tracings. For the analysis during the BCO period, data points from the first 60 s after BCO was initiated (20 s of the BCO period plus the next 40 s after BCO ending) were used. The time series were analyzed in the frequency domain by means of spectral analysis using an open-access custom computer software (CardioSeries v2.7, www.danielpenteado.com). Briefly, the beat-by-beat time series were resampled using cubic spline interpolation (10 Hz), and the interpolated series were split into half-overlapping sequential segments of 512 data points. All segments were visually inspected by a highly experienced researcher looking for transients that could affect the calculation of the power spectral density. To ensure that visual inspection of the time series was properly performed, a Hanning window was used to diminish side effects, and the spectrum was calculated for all segments using a direct Fast Fourier Transform (FFT) algorithm for discrete-time series. Finally, all spectra were visually inspected for abnormalities and were integrated into low- (LF: 0.20–0.75 Hz) and high-frequency (HF: 0.75–3.00 Hz) bands. Results are expressed in absolute (ms^2^ and mmHg^2^) and normalized (nu) units. LF/HF ratio was also calculated. It is worth noting that cardiac interval and arterial pressure variability measured by spectral analysis are useful tools to evaluate the autonomic modulation of the cardiovascular system (32, 33) and have been used in the study of cardiovascular and inflammatory diseases, revealing a strong association between the levels of cytokines and autonomic modulation (34–36). What is more, the HF oscillations of the cardiac interval correspond to the vagal modulation of the heart (37–39) and the LF oscillations of both the cardiac interval and systolic arterial pressure reflect sympathetic modulation of the heart and vessels, respectively (32, 37, 40, 41).

### Cytokine Measurements

Plasma levels of cytokines (TNF, IL-1β, IL-6, and IL-10) were measured by the immune-enzymatic ELISA method using Duo set kits from R&D Systems (Minneapolis, MN, USA) according to the manufacturer’s instructions.

### Statistical Analysis

The results are presented as mean ± standard error of the mean (SEM). The hemodynamic and cardiocirculatory variability parameters were analyzed by two-way analysis of variance (ANOVA) for repeated measurements followed by the Tukey post-test when indicated and also by one-way ANOVA followed by the Tukey post-test when indicated. The data obtained from plasma were analyzed by one-way ANOVA, followed by the Student–Newman–Keuls post-test when indicated and also by the two-way ANOVA for repeated measures followed by the Student–Newman–Keuls post-test. Interrelations between systolic arterial pressure variability and IL-6 and IL-10 levels were examined by Pearson’s correlation after being classified as normally distributed by the Shapiro–Wilk normality test. Interrelations between systolic arterial pressure variability and TNF and IL-1β levels were examined by Spearman’s correlation after not being classified as normally distributed by the Shapiro-Wilk normality test. Differences were considered statistically significant if p < 0.05. Statistical analysis was performed using SigmaPlot 12.0 software (Systat Software, San Jose, CA, USA) and GraphPad Prism 6.0 software (GraphPad Software, San Diego, CA, USA).

## Results

### Hemodynamic and Autonomic Responses to Bilateral Carotid Occlusion

**Figure 1:**
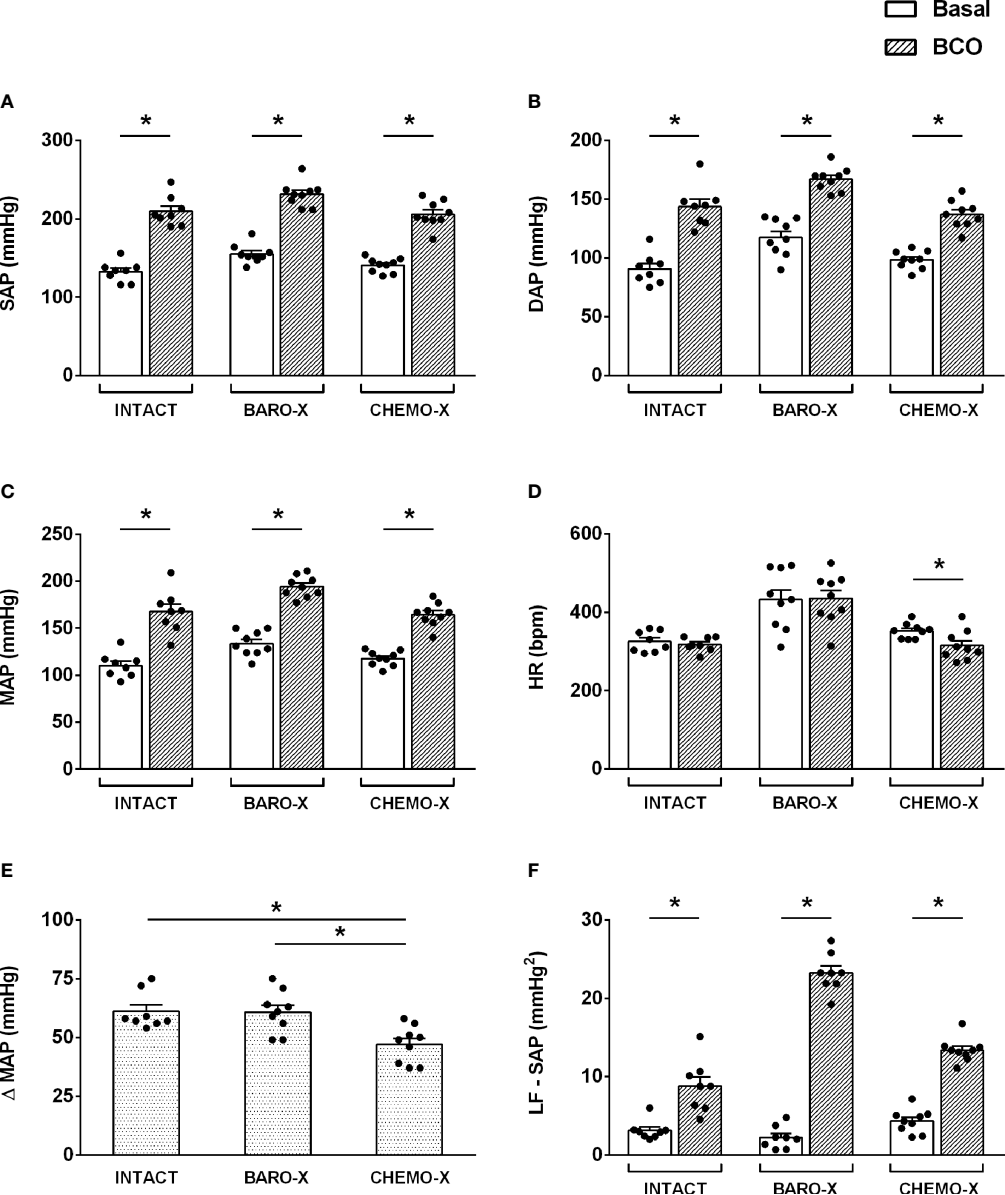
Hemodynamic and autonomic responses to bilateral carotid occlusion (BCO) in intact animals and with denervation of baroreceptors (BARO-X) or chemoreceptors (CHEMO-X). SAP, systolic arterial pressure **(A)**; DAP, diastolic arterial pressure **(B)**; MAP, mean arterial pressure **(C)**; HR, heart rate **(D)**; Δ MAP: the difference between basal and BCO values of MAP **(E)**; LF-SAP: low frequency band in the spectrum of the systolic arterial pressure **(F)**. Panels **(A–D)**: basal period (white bars) and BCO peak response period (period of 5 s within the peak response during BCO; gray bars). Panel **(F)**: basal (white bars) and BCO period (period of 20 s of the BCO plus the next 40 s after BCO ending; gray bars). Bars represent mean ± SEM. *p < 0.05.

BCO promoted an increase in systolic, diastolic, and mean arterial pressure (**Figures 1A–C**), in intact animals and in those with selective denervation of baroreceptors (BARO-X) or chemoreceptors (CHEMO-X), indicating an increase in peripheral resistance due to the sympathetic activation. However, the hypertensive response to BCO was lower in the CHEMO-X than in the intact and BARO-X animals (mean arterial pressure: Intact, Δ 61 ± 3 mmHg; BARO-X, Δ 61 ± 3 mmHg; CHEMO-X, Δ 47 ± 3 mmHg; Intact *vs.* BARO-X, p = 0.998; Intact *vs.* CHEMO-X, p = 0.005; BARO-X *vs.* CHEMO-X, p = 0.005; **Figure 1E**), indicating the importance of the integrity of the chemoreceptors for the peak of hypertensive response during BCO as previously described (26). In addition, while BCO did not change the heart rate of intact and BARO-X animals, it did promote bradycardia in the CHEMO-X subjects (**Figure 1D**), indicating a reflex response involving the aortic baroreceptors activating the parasympathetic function in the heart.

It is worth mentioning that the increase in sympathetic modulation of the vessels, resulting from the BCO maneuver, was in line with the increase of the power of the LF band in the spectrum of the systolic arterial pressure during the BCO period (**Figure 1F**), in both intact and denervated animals (BARO-X and CHEMO-X).

### Time Course of Hemodynamic and Autonomic Responses

**Figure 2:**
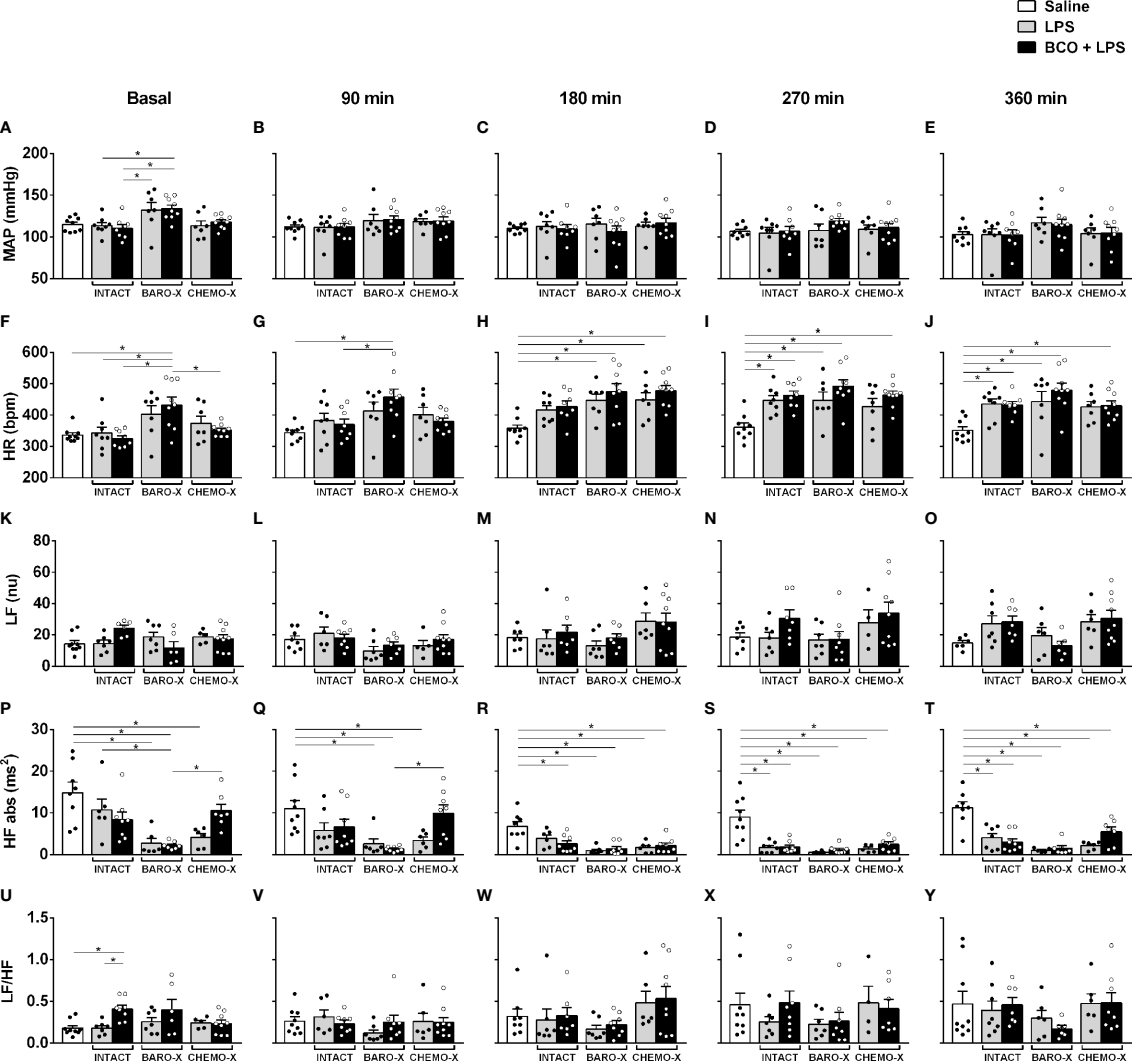
Time course of hemodynamic and cardiac interval variability responses. Mean arterial pressure (MAP; panels **A–E**), heart rate (HR; panels **F–J**), low-frequency band (LF; panels **K–O**), and high-frequency band (HF; panels **P–T**) in the spectrum of the cardiac interval, and LF/HF ratio (panels **U–Y**) at different times: baseline, 90, 180, 270, and 360 min after LPS or saline. BARO-X, selective denervation of baroreceptors; BCO, bilateral carotid occlusion; CHEMO-X, selective denervation of chemoreceptors; LPS, lipopolysaccharide. Bars represent mean ± SEM. *p < 0.05.

The analysis of the hemodynamic parameters from animals with BARO-X showed higher mean arterial pressure than the intact rats under basal conditions (**Figure 2A**). Regarding the other periods evaluated, no difference was observed in the mean arterial pressure among groups at each time frame evaluated (**Figures 2B–E**). Concerning the heart rate under basal conditions, the subjects with BARO-X exhibited higher levels of heart rate than intact—an outcome already described in the literature (42, 43)—and CHEMO-X animals (**Figure 2F**). Ninety min after LPS, the subjects with BARO-X still showed higher heart rate levels than intact rats (**Figure 2G**). Over time, the rats that received LPS showed an increase in heart rate compared to the animals that received saline, starting from 180 min after LPS administration (**Figure 2H**). Moreover, this response was similar at 270 min after LPS injection (**Figures 2I**). In addition, this tachycardia was maintained until the end of the protocol (360 min) in the animals exhibiting endotoxemia (**Figure 2J**).

The analysis of cardiac interval variability did not reveal any difference in the power of the LF band between the groups evaluated over time (**Figures 2K–O**). The same was observed for the LF/HF ratio (**Figures 2U–Y**). Regarding the power of the HF band, a reduction in this parameter was observed in the basal period in the animals with BARO-X compared to the intact control rats (**Figure 2P**). The group with CHEMO-X also showed lower values of the power of the HF band during the basal period (**Figure 2P**). However, the group with CHEMO-X associated with BCO showed an increase in the HF band compared to the BARO-X + BCO + LPS group under basal and 90 min (**Figures 2P, Q**). Over time, the groups that received LPS showed a reduction in the power of the HF band compared to the saline group, as displayed at 180, 270 and 360 min (**Figures 2R–T**).

### Bilateral Carotid Occlusion Reduced Systemic Inflammation

**Figure 3:**
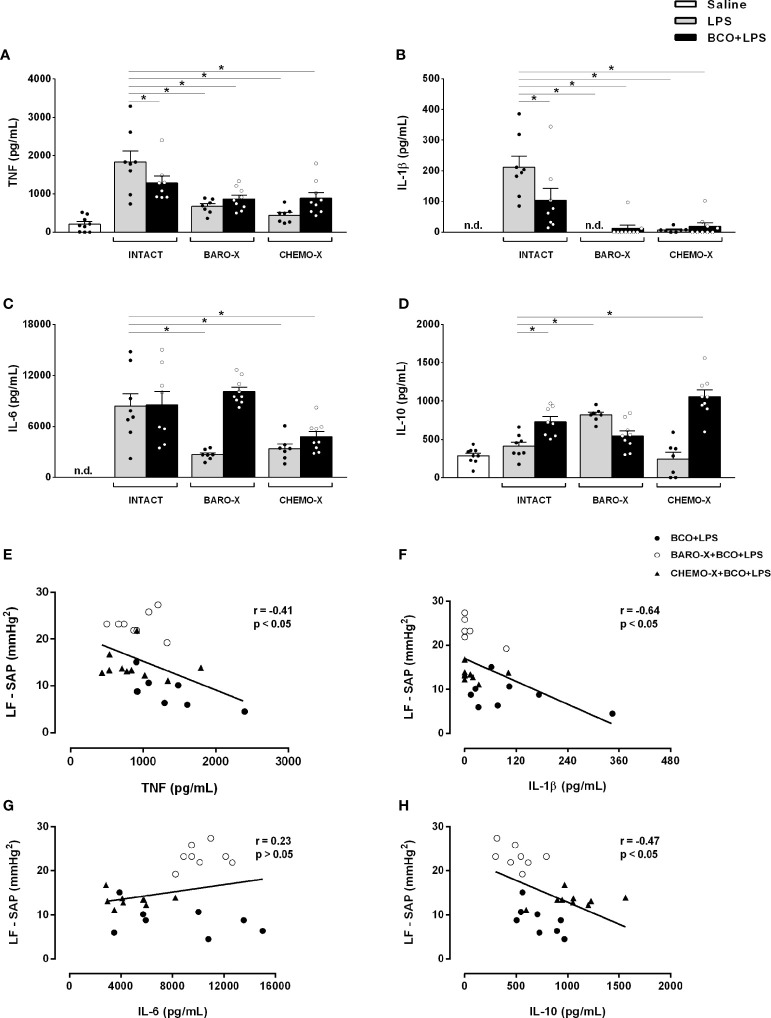
Plasma cytokine levels 90 min after saline or LPS and correlation of systolic arterial pressure variability and cytokine levels. Plasma levels of TNF **(A)**, IL-1β **(B)**, IL-6 **(C)** and IL-10 **(D)** 90 min after administration of LPS or saline, and Pearson’s and Spearman’s correlation between the power of the low-frequency band of the systolic arterial pressure (LF-SAP) during the BCO period and the cytokines plasma levels [TNF **(E)**, IL-1β **(F)**, IL-6 **(G)**, and IL-10 (**H**)] at 90 min after the administration of LPS. BARO-X, selective denervation of baroreceptors; BCO, bilateral carotid occlusion; CHEMO-X, selective denervation of chemoreceptors; LPS, lipopolysaccharide; n.d., not detected. Bars represent mean ± SEM. *p < 0.05.

BCO reduced the plasma levels of TNF (p < 0.001) and IL-1β (p < 0.001) in intact animals 90 min after LPS administration (**Figures 3A, B**, **Table 1**). However, in those animals with BARO-X, BCO did not change the levels of TNF (p = 0.061) or IL-1β (p = 0.946), and in CHEMO-X rats BCO increased TNF levels (p < 0.001), but not modified IL-1β (p = 0.862) 90 min after LPS administration (**Table 1**). For IL-1β, in intact rats, this effect was maintained until 180 min after LPS administration (**Table 1**). In addition, BCO increased the levels of IL-10 in the plasma of intact animals (p = 0.001) at 90 min and those with CHEMO-X up to 270 min (p < 0.001) (**Figure 3D**, **Table 1**). In other words, the anti-inflammatory cytokine levels increased in plasma when the carotid baroreceptors were deactivated since when the BCO was performed in intact and CHEMO-X rats, there was reflexive deactivation of the carotid baroreceptors due to a significant reduction in the arterial pressure and blood flow inside the carotid sinus region. However, in BARO-X animals, the BCO decreased the plasma levels of IL-10 at 90 min (p = 0.007), 270 min (p = 0.049), and 360 min (p = 0.024), but not at 180 min (p = 0.194) (**Table 1**). This data shows that the IL-10 levels decreased in plasma when only the carotid chemoreceptors were activated by BCO. Of note, these results from IL-10 due to BCO performed in different animals (intact, BARO-X and CHEMO-X) confirm that surgical denervation is not the same as the deactivation of a sensory receptor by a physiological mechanism. Finally, BCO was not effective in reducing the plasma IL-6 in intact animals at any of the evaluated time frames (90 min: p = 0.816; 180 min: p = 0.916; 270 min: p = 0.990; 360 min: p = 1) (**Table 1**). Nevertheless, BCO increased IL-6 in BARO-X (p < 0.001) and CHEMO-X (p = 0.037) rats 90 min after LPS administration (**Table 1**). Moreover, for BARO-X subjects this effect was also observed 180 min after LPS (p < 0.001) (**Table 1**).

**Table 1:** Time Course of Plasma Cytokines.

	Saline (n = 9)	LPS (n = 8)	BCO + LPS (n = 8)	BARO-X + LPS (n = 7)	BARO-X + BCO + LPS (n = 9)	CHEMO-X + LPS (n = 7)	CHEMO-X+ BCO + LPS (n = 9)
**TNF (pg/ml)**				
90 min	207 ± 66	1831 ± 290*	1326 ± 180*^#^	671 ± 72*^#^†	873 ± 96*^#^†	442 ± 75*^#^†^$^§	889 ± 146*^#^†‡
180 min	117 ± 36	275 ± 40	206 ± 19	222 ± 15	299 ± 21	69 ± 9	213 ± 22
270 min	105 ± 25	120 ± 18	100 ± 8	114 ± 18	226 ± 18	30 ± 7	121 ± 8
360 min	89 ± 20	99 ± 12	76 ± 5	92 ± 14	181 ± 12	24 ± 8	97 ± 9
**IL-6 (pg/ml)**
90 min	0 ± 0	8389 ± 1480*	8546 ± 1560*	2684 ± 225*^#^†	10120 ± 502*^#^†^$^	3398 ± 533*^#^†§	4810 ± 587*^#^†^$^§‡
180 min	0 ± 0	2977 ± 588*	3048 ± 1191*	252 ± 92^#^†	3888 ± 695*^$^	0 ± 0^#^†§	398 ± 49^#^†§
270 min	0 ± 0	14 ± 12	22 ± 19	0 ± 0	0 ± 0	0 ± 0	0 ± 0
360 min	0 ± 0	0 ± 0	0 ± 0	0 ± 0	0 ± 0	0 ± 0	0 ± 0
**IL-1β (pg/ml)**
90 min	0 ± 0	212 ± 35*	104 ± 39*^#^	0 ± 0^#^†	12 ± 11^#^†	7 ± 3^#^†	19 ± 11^#^†
180 min	2 ± 1	178 ± 26*	116 ± 50*^#^	18 ± 12^#^†	0 ± 0^#^†	4 ± 2^#^†	19 ± 11^#^†
270 min	0 ± 0	82 ± 18*	47 ± 20	0 ± 0^#^	23 ± 14^#^	0 ± 0^#^	8 ± 5^#^
360 min	0 ± 0	39 ± 35	11 ± 7	0 ± 0	2 ± 1	0 ± 0	3 ± 2
**IL-10 (pg/ml)**				
90 min	287 ± 34	410 ± 55	730 ± 66*^#^	819 ± 33*^#^	546 ± 63*†^$^	245 ± 86†^$^§	1057 ± 88*^#^†^$^§‡
180 min	198 ± 18	474 ± 51*	523 ± 56*	989 ± 173*^#^†	730 ± 70*^#^†	307 ± 94^$^§	900 ± 108*^#^†‡
270 min	105 ± 12	229 ± 38	295 ± 39	539 ± 49*^#^†	328 ± 40*^$^	14 ± 13†^$^§	349 ± 48*^$^‡
360 min	43 ± 11	122 ± 31	137 ± 13	415 ± 50*^#^†	222 ± 42^$^	0 ± 0^$^	188 ± 29^$^

Data are expressed as mean ± SEM. TNF, tumor necrosis factor; IL-6, interleukin 6; IL-1β, interleukin 1β; IL-10, interleukin 10. *p < 0.05 vs. Saline at the same moment; ^#^p < 0.05 vs. LPS at the same moment; † p < 0.05 vs. BCO + LPS at the same moment; ^$^p < 0.05 vs. BARO-X + LPS at the same moment; § p < 0.05 vs. BARO-X + BCO + LPS at the same moment; ^‡^p < 0.05 vs. CHEMO-X + LPS at the moment. BARO-X, selective denervation of baroreceptors; BCO, bilateral carotid occlusion; CHEMO-X, selective denervation of chemoreceptors; LPS, lipopolysaccharide.

### Denervation of the Baroreceptors or Chemoreceptors Modulates Systemic Inflammatory Response

Surprisingly, the surgical denervation of the aortic and carotid baroreceptors attenuated the release of TNF, IL-6, and IL-1β into the plasma 90 min after LPS administration (**Figures 3A–C**, **Table 1**). This effect remained until 180 min after triggering the immune challenge for IL-6, and until 270 min for IL-1β (**Table 1**). The same outcome was observed in animals that underwent CHEMO-X (**Figures 3A–C**, **Table 1**), suggesting a possible communication among both baroreceptors and chemoreceptors that modulated the inflammatory response. Considering the plasma levels of IL-10, compared to the LPS group, the BARO-X stimulated its release during the basal period (**Table 1**) and maintained the same response 90 min after the administration of LPS (**Figure 3D**, **Table 1**), as well as in the other periods evaluated (**Table 1**). On the other hand, no difference was observed in IL-10 plasma levels in any time frame periods in the CHEMO-X + LPS group compared to the LPS group (**Table 1**).

### Correlation of Sympathetic Modulation and Cytokines Levels Release

Negative correlations between the sympathetic modulation and TNF (**Figure 3E**), IL-1β (**Figure 3F**) and IL-10 (**Figure 3H**) plasma levels were found. These results can be interpreted as follows: the higher the reflex sympathetic modulation, the lower the cytokine release in unanesthetized endotoxemic rats. In contrast, no correlation was found between the sympathetic modulation and IL-6 plasma level (**Figure 3G**).

## Discussion

The present study shows for the first time that a reflex physiological activation of the sympathetic nervous system reduces the systemic LPS-induced inflammatory response. In addition, it was also demonstrated that surgical denervation of the aortic and carotid baroreceptors, as well as of the peripheral chemoreceptors, by themselves decrease the plasma cytokines levels in endotoxemic rats.

Previous studies have highlighted the role of the autonomic nervous system branches controlling the immune system (6–12). However, taking into account that the body uses fine regulatory mechanisms to preserve homeostasis, this is the first study showing that a conspicuous physiological reflex activation controls an inflammatory response. The BCO technique promotes a reduction of the perfusion pressure and the blood flowing into the carotid sinus region, culminating, therefore, in the deactivation of the carotid baroreceptors and activation of the peripheral chemoreceptors. These outcomes determine the increase in sympathetic activity, particularly upon the arterioles, increasing the global peripheral resistance, combined with a concomitant reduction in parasympathetic activity to the heart, thus increasing arterial pressure (26, 27).

The BCO promoted an increase in arterial pressure in both intact and denervated (BARO-X or CHEMO-X) groups. However, compared to the baseline values, BCO promoted a smaller rise of the mean arterial pressure in the CHEMO-X than with the intact and BARO-X animals. Overall, in intact animals, when the common carotid arteries are occluded temporarily, there is the inactivation of the carotid baroreceptors and the carotid activation chemoreceptors contribute to an increase in sympathetic activity. When the BCO is performed in the absence of peripheral chemoreceptors (CHEMO-X), there is only the effect from the inactivation of carotid baroreceptors, which can partly explain the smaller increase in mean arterial pressure in the CHEMO-X compared to other groups. Moreover, this data suggests that chemoreflex activation plays a more significant role in increasing arterial pressure during BCO than baroreflex deactivation. Regarding the heart rate, the effect promoted by BCO was different between the groups. Intact rats and those with denervated baroreceptors did not show a change in heart rate. In contrast, the animals with denervated chemoreceptors exhibited bradycardia during BCO. We have to consider that during BCO in the intact animals there is a combination of the different mechanisms at the same time (1. activation of peripheral chemoreceptors; 2. deactivation of carotid baroreceptors; and 3. activation of aortic baroreceptors). This combination of mechanisms, especially involving central interaction, can result in many responses. In this case, no change in heart rate. In the CHEMO-X group, the decrease in heart rate during BCO could be due to a reflex response involving the aortic baroreceptors induced by the increase in arterial pressure, activating the parasympathetic branch to the heart. In the BARO-X group, the chemoreflex activation during BCO may have augmented the sympathetic activation to the vessels, increasing the arterial pressure, but it may not have been enough to change the heart rate.

In the present study, the sympathetic activation elicited by BCO decreased pro-inflammatory plasma cytokines (TNF and IL-1β) and increased the anti-inflammatory cytokine (IL-10) in intact rats, contributing to the control of the systemic inflammatory response. Moreover, a negative correlation was found between the sympathetic modulation, assessed by the power of the LF band of systolic arterial pressure spectra and cytokine production, suggesting that the higher the sympathetic modulation of the resistance vessels during the BCO, the lower the pro-inflammatory cytokine release induced by LPS. Of note, an increase in the sympathetic modulation of the resistance vessels during the BCO approach was shown by the rise of the power of the LF band of the systolic arterial pressure spectra, since this parameter is related to the peripheral resistance from blood vessels (41). Thus, taking into account that BCO determines a significant sympathetic activation, and given that the spleen, an organ known as the main source of cytokines, is innervated by the sympathetic nervous system, it is suggested that BCO can stimulate the celiac ganglion, inhibiting the release of pro-inflammatory cytokines by the splenic macrophages (4, 11, 44).

The selective denervation of aortic and carotid baroreceptors reduced the plasma levels of TNF and IL-6 compared to intact animals (LPS group). Moreover, the IL-1β release induced by LPS was abolished in animals without baroreceptors. The same response was observed in animals that had only their carotid chemoreceptors denervated. These exciting data suggest that both baroreceptors and chemoreceptors have an essential role in the signaling of the inflammatory response in endotoxemic rats, favoring the release of cytokines. Regarding the role of the chemoreceptors in the inflammatory response, several studies have shown that glomus cells from the carotid body have receptors for pro-inflammatory cytokines (TNF, IL-6, and IL-1), expressing TLR-4, responsible for LPS recognition (45–51). Moreover, the administration of LPS increased the expression of TNF and TNF receptor in the carotid body, increasing the immune response (46, 47). Thus, the findings of the current study, as well as those from the literature (47) suggest an immunosensory function of the carotid body as a peripheral sensor for the presence of immunogenic agents from the blood.

Little is known about the role of the baroreceptors influencing the signaling of the inflammatory response. Since some studies have proposed that sympathetic neurons are the efferent arm of the inflammatory reflex (52) and the activation of the baroreceptors promotes inhibition of the sympathetic nervous system (53), it is plausible to propose that in the absence of the baroreceptors the sympathetic nervous system would act on the spleen, inhibiting the release of cytokines by the splenic macrophages. In addition, it is also known that both TLR-4 and cytokine receptors are expressed in neurons and the nodose ganglion (54–58), suggesting, therefore, that LPS and peripheral cytokines could inform the brain about a peripheral inflammatory response *via* baroreceptor nerve endings. Moreover, a recent study showed that sinoaortic denervation attenuated the release of plasma IL-6 and IL-10 in endotoxemic rats (59). However, since sinoaortic denervation includes the removal of the carotid chemoreceptors (30, 31, 60, 61), it is possible that the response observed in the aforementioned study occurred due to the absence of the chemoreceptors, but not baroreceptors. Nevertheless, it is clear that the baroreceptors play a role in modulating the immune response, and additional investigations of the mechanism involved are required.

In animals that had their baroreceptors denervated, the IL-1β release induced by LPS was abolished. In rats subjected to the carotid chemoreceptors’ denervation, there was also a substantial reduction of this cytokine in plasma compared to the LPS group (intact animals). The IL-1β release depends on the process intermediated by caspase-11 and the NLRP3 inflammasome in which the cytokine precursor pro-IL-1β is cleaved into mature IL-1β. Of note, two steps are needed for NLRP3 inflammasome activation: (1) a priming step, which is provided by an inflammatory stimulus such as TLR-4 agonists, and (2) an activation step, which is stimulated by pathogen-associated molecular patterns (PAMPs) and danger-associated molecular patterns (DAMPs) (62). However, intracellular LPS can also activate the NLRP3 inflammasome by the caspase-11 route and stimulate IL-1β release (18, 19). Thus, the absence of baroreceptors or chemoreceptors may affect both the caspase-11/NLRP3 pathway, which is responsible for releasing the mature IL-1β, and the transcription of pro-IL-1β by TLR-4 stimulus, contributing together to the reduction of this cytokine in plasma. A limitation of our study has to be mentioned. Given that we did not use ultrapure LPS isolated from *Escherichia coli*, some contaminants could activate other receptors from TLR-4 and release the cytokines investigated.

Over time, the administration of LPS decreased the power of the HF band, but not the LF band, in the cardiac interval spectrum. Likewise, none of the techniques used in the present study (BCO, BARO-X or CHEMO-X) changed the power of the LF band in the spectrum of the cardiac interval over time. Of note, the groups with BARO-X already showed a decrease in the power of the HF band of the cardiac interval spectrum in the baseline period, confirming a reduction of vagal modulation when the aortic and carotid baroreceptors are absent. Corroborating previous studies (59, 63, 64), the data from the current study indicate that during systemic inflammation, the absence of the aortic and carotid baroreceptors, or the peripheral chemoreceptors, does not significantly affect the sympathetic modulation of the heart, but decreases its vagal modulation. It is essential to highlight that the analysis of cardiocirculatory variability is a remarkable tool for understanding the autonomic modulation of the heart and vessels in a number of situations (41, 65, 66). Moreover, the assessment of the autonomic balance is an important analytical tool that is reliably useful under different clinical conditions, including infectious and autoimmune diseases (67, 68). Furthermore, the heart rate variability parameters are used for the diagnosis and monitoring of patients with sepsis (69, 70). Furthermore, this non-invasive approach does not promote additional stress and hemodynamic alterations in patients and experimental animals.

In conclusion, these results show, for the first time in the literature, that the reflex physiological activation of the sympathetic circuit decreases the inflammatory response in endotoxemic rats. In addition, the data indicate that the baroreceptors (aortic and carotid) and the peripheral chemoreceptors contribute to the development of the systemic inflammatory response induced by LPS since their absence attenuates the release of pro-inflammatory cytokines.

## Data Availability Statement

The raw data supporting the conclusions of this article will be made available by the authors without undue reservation.

## Ethics Statement

The animal study was reviewed and approved by Committee of Ethics in Animal Research of the Ribeirão Preto Medical School - University of São Paulo (Protocol # 194/2016).

## Author Contributions

FB conceived and designed the research study, performed experiments, analyzed data, interpreted results, prepared figures and the table, and wrote the manuscript. JC performed experiments and interpreted results. AK interpreted the results, commented and edited the manuscript. DD analyzed data, commented and edited the manuscript. HS conceived and designed the research study, commented and edited the manuscript. All authors contributed to the article and approved the submitted version.

## Funding

Supported by The São Paulo Research Foundation (FAPESP) process #2013/20549-7 and #2017/05163-6, by The Academic Excellence Program (PROEX) from Coordination for the Improvement of Higher Education Personnel (CAPES) process #88887.505419/2020-00, and by Foundation to Support Teaching, Research, and Assistance (FAEPA).

## Conflict of Interest

The authors declare that the research was conducted in the absence of any commercial or financial relationships that could be construed as a potential conflict of interest.
