# Supplementary material for: Physiological Sympathetic Activation Reduces Systemic Inflammation: Role of Baroreflex and Chemoreflex
Source: Front Immunol. 2021 Apr 28;12:637845. doi: 10.3389/fimmu.2021.637845 (PMC8117744; doi:10.3389/fimmu.2021.637845)
Supplement: Supplementary file 1 [file Image_1.pdf]

Supplementary Material

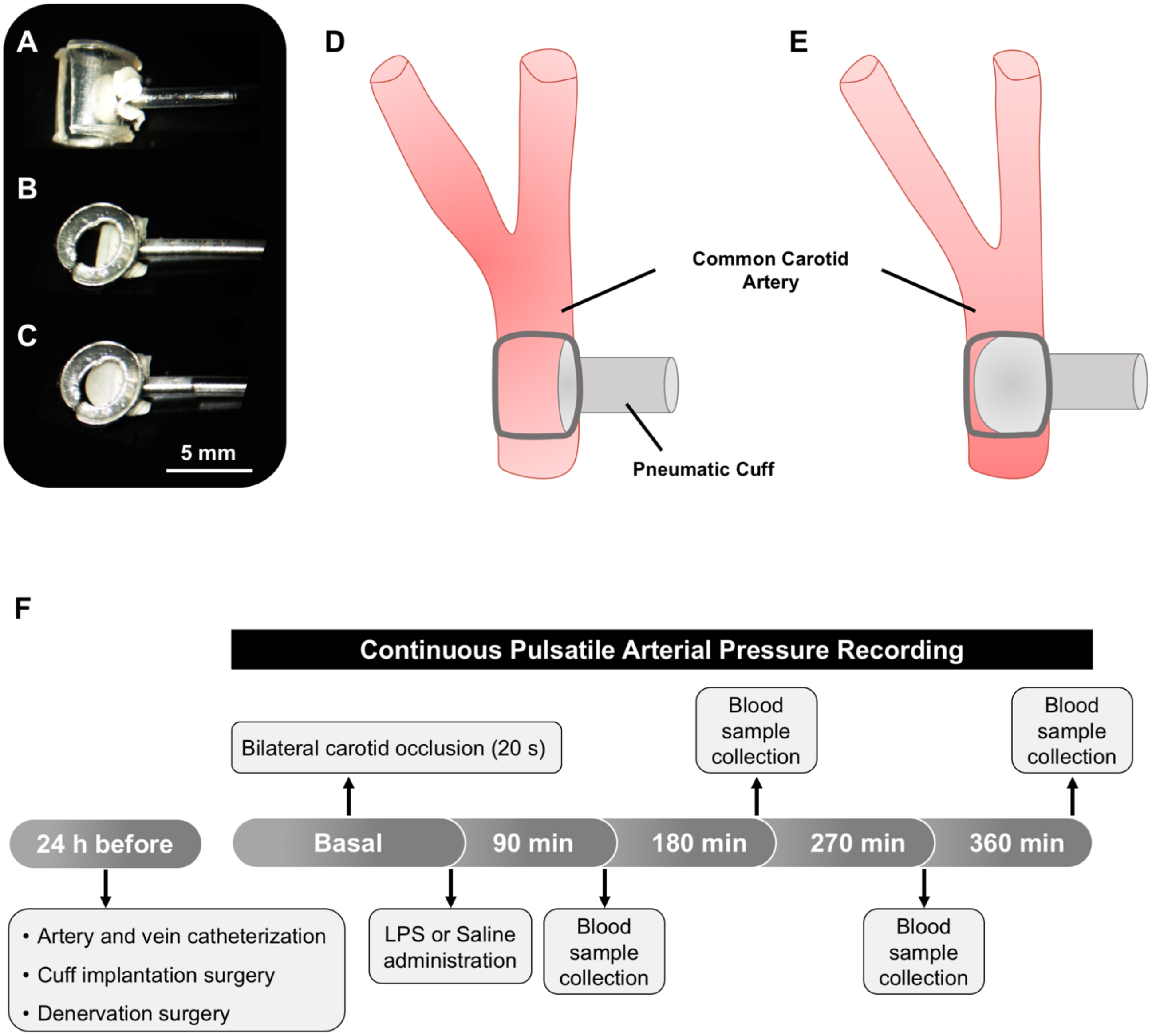

Supplementary Figure 1. The pneumatic cuff, a schematic representation of the method used, and the experimental protocol. A: lateral view of the pneumatic cuff; B: pneumatic cuff with the balloon without inflation; C: pneumatic cuff filled with water inflating the balloon. D: schematic representation at the basal period, when there was no occlusion of the common carotid artery (normal blood flow). E: schematic representation during the carotid occlusion. The balloon inside the pneumatic cuff was inflated with water using a syringe with an unsharpened needle connected to the polyethylene tube. Note that the blood flow, as well as the diameter of the internal and external carotid arteries, are reduced in the region above the occlusion site, deactivating the carotid baroreceptors. F: the timeline of the experimental protocol used in the present study.
